# Metabolic Disturbance in PCOS: Clinical and Molecular Effects on Skeletal Muscle Tissue

**DOI:** 10.1155/2013/178364

**Published:** 2013-06-05

**Authors:** Wagner Silva Dantas, Bruno Gualano, Michele Patrocínio Rocha, Cristiano Roberto Grimaldi Barcellos, Viviane dos Reis Vieira Yance, José Antonio Miguel Marcondes

**Affiliations:** ^1^School of Physical Education and Sport, Laboratory of Applied Nutrition and Metabolism, University of São Paulo, 05508-030 São Paulo, SP, Brazil; ^2^Endocrinology Division, School of Medicine, University of São Paulo, 05508-030 São Paulo, SP, Brazil

## Abstract

Polycystic ovary syndrome is a complex hormonal disorder affecting the reproductive and metabolic systems with signs and symptoms related to anovulation, infertility, menstrual irregularity and hirsutism. Skeletal muscle plays a vital role in the peripheral glucose uptake. Since PCOS is associated with defects in the activation and pancreatic dysfunction of *β*-cell insulin, it is important to understand the molecular mechanisms of insulin resistance in PCOS. Studies of muscle tissue in patients with PCOS reveal defects in insulin signaling. Muscle biopsies performed during euglycemic hyperinsulinemic clamp showed a significant reduction in glucose uptake, and insulin-mediated IRS-2 increased significantly in skeletal muscle. It is recognized that the etiology of insulin resistance in PCOS is likely to be as complicated as in type 2 diabetes and it has an important role in metabolic and reproductive phenotypes of this syndrome. Thus, further evidence regarding the effect of nonpharmacological approaches (e.g., physical exercise) in skeletal muscle of women with PCOS is required for a better therapeutic approach in the management of various metabolic and reproductive problems caused by this syndrome.

## 1. Introduction

Polycystic ovary syndrome (PCOS) is one of the most common endocrine disorders, affecting approximately 5–7% of women in reproductive age [1]. It was first described by Stein and Leventhal in 1935, who found an association between amenorrhea, hirsutism, and obesity with polycystic ovaries. The authors reported on bilaterally enlarged ovaries, with a thick and whitened capsule [2], multiple cysts located mainly in the subcapsular region, and a hypertrophied stroma.

Subsequently, the heterogeneity of the clinical features led to the adoption of the term “polycystic ovary syndrome.” Following the introduction of new investigative techniques, such as hormone measurements by radioimmunoassay and ovarian morphology by ultrasound, the earlier diagnosis diagnosis based only on clinical and anatomical criteria was replaced by a new one which incorporates hormonal and ultrasonographic criteria [3].

Considered by the end of the last century as a disorder of the reproductive system (given the presence of menstrual disturbance and consequent infertility) and with aesthetic repercussion (given the presence hyperandrogenism, hirsutism, acne, and alopecia), nowadays the syndrome is also considered an important cardiovascular risk factor [4].

In fact, there is evidence of early impairment of the vascular system. Methods which determine the presence of subclinical atherogenesis, such as the endothelial function assessment, which measures the intima-media thickness of the carotid artery and the arterial compliance of the brachial artery were used in some studies [5]. Although not universally documented, vascular damage was observed in patients with PCOS compared with women without the syndrome. More recently, it was shown that postmenopausal patients with previous history of the syndrome have, when undergoing coronary catheterization, experienced a greater number of lesions and a worse prognosis after catheterization [6].

Some conditions may be associated with PCOS, such as endometrial hyperplasia and carcinoma, obesity carbohydrate intolerances, type 2 diabetes, lipid metabolism disorders, hypertension and sleep apnea. Importantly, all of these conditions are associated with an increased long-term risk for cardiovascular disease. A possible link between these conditions and cardiovascular disease is insulin resistance, which is present regardless of body mass index, but potentialized by obesity [7]. It was recently documented an impaired cardiopulmonary functional capacity strictly related to insulin resistance in women with the syndrome [8]. In order to standardize the diagnosis of PCOS, various guidelines and statements have been published in recent years, resulting in the combination of the fundamental characteristics of the syndrome, that is hyperandrogenemia (increase in testosterone and/or DHEAS concentration), hyperandrogenism (hirsutism, acne, or alopecia), menstrual dysfunction, and polycystic ovarian morphology identified by ultrasound.

**Figure 1:**
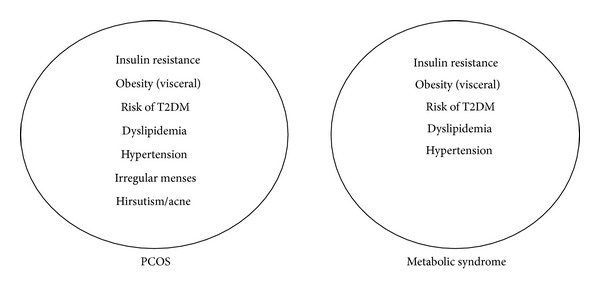
Common features of PCOS and the metabolic syndrome. Adapted from Tfayli and Arslanian [14].

The three most frequent consensus are shown in Figure 1 and Table 1. A consensus on these guidelines is that PCOS is a syndrome and not a specific disease. Consequently, no single criterion can define its diagnosis, therefore it is a diagnosis of exclusion.

## 2. Metabolic Syndrome and PCOS

MetS is a cluster of metabolic abnormalities, primarily abdominal obesity, insulin resistance, compensatory hyperinsulinemia, impaired glucose metabolism, dyslipidemia, inflammation, endothelial dysfunction, and hypertension that currently affects approximately one out of five women in reproductive age [16]. In addition, several prospective studies have shown that MetS is associated with an increased risk for type 2 diabetes mellitus and subclinical and clinical cardiovascular diseases [14]. MetS shares many similarities with PCOS, including the frequent presence of abdominal obesity and insulin resistance [14]. PCOS is now considered as a female subtype of the metabolic syndrome, and its potential health consequences have been considered as a public-health concern (Figure 1).

The prevalence of MetS in women with PCOS largely varies, from 1.6 to 43% depending on assessed population [17–19]. The prevalence of MetS in PCOS patients was evaluated in a study conducted in the city of São Paulo (Brazil). Seventy-three women, with body mass index (BMI) of 30.4 ± 7.8 kg/m^2^ and 25.0 ± 6.0 years, subdivided according to BMI, were studied retrospectively. According to the modified criteria of the Third Report of the National Cholesterol Education Program (NCEP/ATP III) for the diagnosis of MetS, which was replaced by the fasting glycemia and glycemia at 120 minutes obtained from oral glucose tolerance test, the prevalence of MetS was 85.5% in those with BMI ≥ 40 kg/m^2^, 62.9% in those with BMI between 30 and 39.9 kg/m^2^, 23.8% in those with overweight, BMI between 25.0 and 29.9 kg/m^2^, and 0% in patients with BMI < 25 kg/m^2^. In this study, the abdominal circumference greater than 88 cm was considered one of the best predictors for the MetS [19].

**Figure 2:**
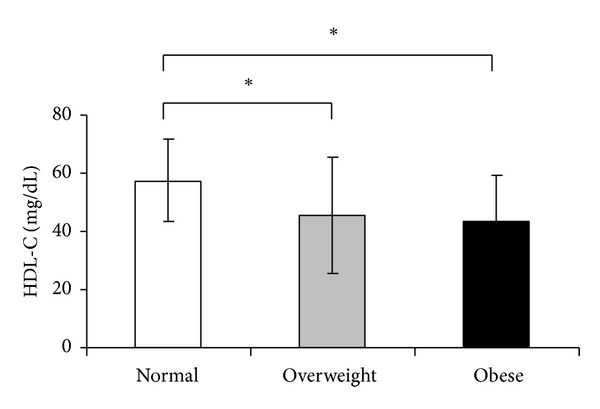
The serum HDL-C level (mean ± SD), according to the BMI. *P < 0.05 [15].

Dyslipidemia in PCOS is multifactorial and appears to be mediated by insulin resistance and androgen excess as well as environmental factors. In PCOS, a number of lipid abnormalities has been found. The most frequent is a decrease in HDL-C and an increase in triglycerides, which is a lipid pattern known to be associated with insulin resistance. Obese women with PCOS have the most atherogenic lipid profiles [20, 21]. Rocha et al. (2011) studied one hundred forty-two women with PCOS with an average BMI of 29.1 kg/m^2^ and an average age of 25.12 years. According to the BMI, 30.2% were normal weight, 38.0% were overweight, and 31.6% were obese. Thirty-one eumenorrheic women matched for BMI and age, with no evidence of hyperandrogenism, were recruited as controls. The incidence of dyslipidemia in the PCOS group was twice that of the control group (76.1% versus 32.25%). The most frequent abnormalities were low HDL-C (57.6%) and high triglyceride (28.3%). HDL-C was significantly lower in all subgroups of healthy with PCOS when compared to the subgroups of healthy women, and the BMI had a significant impact on this abnormality [15] (Figure 2).

## 3. Impaired Glucose Tolerance and Type 2 Diabetes Mellitus in PCOS

The prevalence of insulin resistance (IR) in PCOS patients have ranged from 44 to 70% [22–26]. This wide range may be due to several factors, including the heterogeneity of the diagnostic criteria for PCOS employed in these studies [22], the genetic background among the assessed population [6], and the differences regarding the methods used for defining IR [22, 25, 26]. It has been shown that the presence of chronic anovulation associated with higher androgen levels was associated with lower insulin sensitivity and higher prevalence of cardiovascular risk factors, such as IR, impaired glucose tolerance (IGT), type 2 diabetes mellitus, and dyslipidemia [22], However the presence of two PCOS phenotypes identified according to the Rotterdam criteria—hyperandrogenism and polycystic ovaries with ovulatory cycles and anovulation and polycystic ovaries without hyperandrogenism—have little or no evidence for IR using surrogate markers [22]. Regarding the ethnicity, there is evidence suggesting that insulin sensitivity may be determined by genetic factors. Goodarzi et al. [27] showed that Mexican-Americans PCOS patients have higher incidence of IR when compared with other ethnic groups.

There are several methods for detecting IR, such as the hyperinsulinemic-euglycemic clamp technique, the fasting insulin, the homeostatic model assessment of IR (HOMA-IR), the quantitative insulin sensitivity check index (QUICKI), the area under the curve of insulin, and the frequent sample IV glucose tolerance test (FSIVGTT). It is known that these methods differ with respect to their accuracy in assessing IR but no study involving PCOS has demonstrated that the incidence of IR depends on the IR assessment method [29]. Even normal weight PCOS patients may suffer from IR [30]. Nonetheless, it is known that both PCOS and obesity have an additive deleterious effects on insulin sensitivity and its metabolic complications [30–32].

Given the frequent occurrence of IR in PCOS, it is not surprising that PCOS is associated with impaired glucose tolerance (IGT) and type 2 diabetes mellitus (T2DM), and the syndrome is now considered to be a significant risk factor for development of T2DM [21]. Up to 35–40% of women with PCOS have IGT, and 10% develop T2DM during the third or fourth decade of life [33–35]. Moreover, epidemiologic studies indicate that the odds ratio for the development of diabetes in women with PCOS is around 2.0 after adjusting for BMI. By amplifying insulin resistance, is a confounding factor in the development of IGT and T2DM in women with PCOS, but the increasing prevalence of obesity in the population means that a further increase in the prevalence of diabetes is also expected [21].

**Figure 3:**
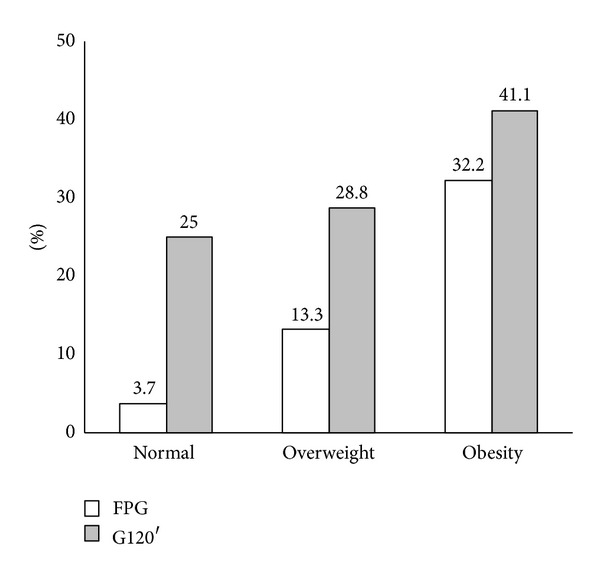
Prevalence of disorders of carbohydrate metabolism in patients with PCOS according to the BMI [28].

The study conducted by Barcellos et al. (2007) showed that the prevalence of disorders of carbohydrate metabolism (i.e., impaired fasting glucose, IGT, and T2DM) in patients with PCOS, using the fasting plasma glucose (FPG) and the plasma glucose at 120 minutes after a challenge with 75 grams of glucose (G120′) in the oral glucose tolerance test. In this study, the normality criteria employed for FPG and G120′ were <100 mg/dL and <140 mg/dL, respectively. Patients were subdivided into three groups according to BMI as follows: normal BMI, overweight, and obese. Using FPG and G120′, the prevalence of IR observed in women with normal BMI, overweight and obesity were 3.7%, 13,3% and 32,2%, respectively, (Figure 3). That is, the prevalence of intolerance to carbohydrate was progressively higher with the increasing BMI, regardless of diagnostic criteria employed. One of the conclusions of this study was that all PCOS patients should be tested with oral glucose tolerance, since this method was more sensitive than FG in the detection of IR [28].

## 4. Insulin Signaling in Skeletal Muscle with PCOS Women

Skeletal muscle plays a pivotal role in the peripheral glucose uptake. In healthy subjects with normal weight, almost one-third of the ingested glucose is taken up by the liver after a meal whilst almost two-third is taken up by skeletal muscle through insulin-dependent mechanisms. After the glucose intake, the increase in plasma glucose stimulates insulin secretion via pancreatic beta cells. Increased insulin resulting from increased plasma glucose suppresses lipolysis decreasing the rate of lipid oxidation [36]. Simultaneously, insulin stimulates glucose uptake by skeletal muscle, increasing the glucose outflow, and by activation of enzymes related to glucose oxidation in this site [37]. 

**Figure 4:**
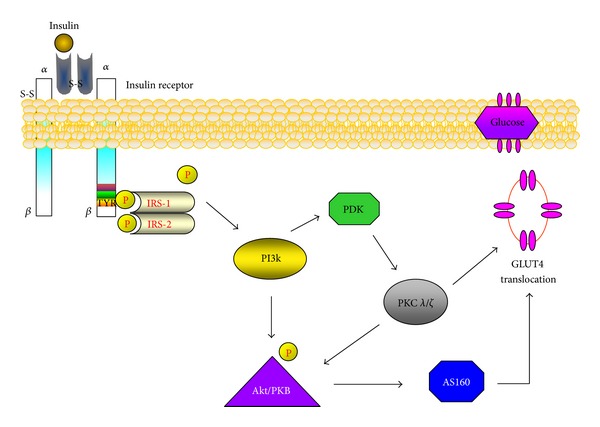
In brief, the insulin binds with its membrane receptor which has intrinsic tyrosine kinase activity, triggers a signaling cascade to downstream substrates resulting in glucose transport. Subsequently, tyrosine phosphorylated IRS (IRS-1/2) recruits signaling molecules incluinding phosphoinositide 3-kinase (PI3k). After a activation of PI3k a complex formation ofphosphatidylinositol-3,4,5-trisphosphate (PI3P) that serves as regulator of phosphoinositide-dependent kinase (PDK) which was later shown to activate others prototypes proteins kinase (e.g., PKC). With this, the protein Akt is activated and propagates the hormonal signal to activate protein AS160 (GTPase activating protein of 160 kDa), which in turn sensitizes the glucose transporter in skeletal muscle (GLUT-4) to the translocation process to the lipid membrane to glucose uptake [38].

The cellular events that initiate the crosstalk between insulin and its receptors are present in the specific surface of skeletal muscle cells. The insulin receptor consists of two subunits (*α* and β) linked by disulfide bonds lying in the extracellular environment sarcoplasmic membrane. The binding of insulin with its receptor leads to phosphorylation of the β-subunit in several tyrosine residues as the insulin receptor has kinase activity [39]. However, due to the hydrophilic characteristic of the glucose molecule, it does not diffuse through the lipid layer of cell membrane. Therefore, it is necessary a membrane transporter to make possible the uptake of glucose by the cell. In humans, these proteins constitute a family of transporters (GLUT) [39]. GLUT-4 express is the major transporter in skeletal muscle, activated (and translocated) to the surface of the cellular membrane in response to insulin and exercise [40–42]. The GLUT-4 translocation is stimulated by insulin in skeletal muscle and the reduced speed-determining step in the glycogen synthesis are observed in T2DM patients [43]. While evidence suggests impairment in the GLUT-4 translocation in patients with T2DM, the total GLUT-4 content is not reduced in the skeletal muscle of type 2 diabetic patients [43]. Therefore, the uptake of glucose into skeletal muscle in insulin-resistant individuals can be partially explained by defects in insulin signaling in the GLUT-4 translocation [44]. An overview of the insulin signaling pathways regulating glucose transport can be seen in Figure 4.

Since PCOS is associated with defects in insulin activation and β-cell pancreatic dysfunction [45], the interest in the molecular mechanisms underlying the insulin resistance in PCOS has increased. Insulin resistance in the skeletal muscle is a major risk factor for the development of T2DM in women with PCOS [46]. For instance, Dunaif et al. (1995) studied skeletal muscle tissue of obese and lean PCOS and and reported an excessive serine phosphorylation (Ser^312^) of insulin receptor in cultured human muscle cells and fibroblasts [47]. However, Corbould et al. (2005) did not confirm these previous findings in cultured skeletal muscle of obese women with PCOS, showing a decrease in insulin sensitivity in cultured muscle cells from women with PCOS, but normal basal phosphorylation levels as well as normal phosphorylation of tyrosine β-subunit of the insulin receptor after stimulation with insulin [48].

Muscle biopsies performed during hyperinsulinemic euglycemic clamp showed that a significant reduction in glucose uptake mediated by insulin and IRS-2 significantly increased in skeletal muscle. In the basal period, the activity of IRS-1-associated phosphoinositide 3-kinase (PI3k) was shown to be normal, but insulin-mediated activity of IRS-1-associated PI3k was significantly reduced [49]. The increased expression of IRS-2 protein in skeletal muscle in women with PCOS may be interpreted as a potential compensatory mechanism of the decreased insulin sensitivity. Yet, the attenuated insulin sensitivity (as assessed by the hyperinsulinemic euglycemic clamp) suggests that protein expression of IRS-2-associated PI3k cannot compensate this decreased sensitivity [45]. Evidence of defects in the post-receptor insulin signaling has been shown *in vivo* in women with PCOS. The basal phosphorylation levels of Akt at Ser^473^ and Thr residues^308^are not altered in women with PCOS women compared with controls [50]. However, when the group of women with PCOS was subjected to an euglycemic hyperinsulinemic clamp, phosphorylation at both residues was attenuated independently of obesity [51]. The total amount of protein TBC1D4 (also known as AS160) in skeletal muscle of women with PCOS is not different at baseline compared to control women. Nonetheless, the phosphorylation of TBC1D4 in women with PCOS undergoing biopsies hyperinsulinemic euglycemic clamp was attenuated compared to control women [52].

Several pharmacological options for attenuating IR are available. Thiazolidinediones (TZDs) are agonists of the peroxisome proliferator-activated receptor (PPAR *γ*). Pioglitazone (one of the main representatives drugs of this class) exerts its effect through mechanisms related to the expression of genes involved in mitochondrial biogenesis, insulin signal transduction, and glucose and lipid metabolism [53]. The PPAR *γ* is abundantly expressed in adipose tissue, and to a lesser extent, in liver and muscle tissue [54]. Women with PCOS treated with pioglitazone (30 mg per day) showed improved insulin sensitivity and a decreased insulin secretion [55]. The molecular mechanisms of the beneficial action of TZDs in skeletal muscle tissue are not fully understood, but they may include increased insulin receptor downstream signaling [56] and improved the uptake and oxidation of free fatty acids [57]. Treatment with TZDs is also associated with increased activity of AMP-activated protein kinase (AMPK) and PPAR *γ* coactivator-1-*α* (PGC-1-*α*) in skeletal muscle [58].

In conclusion, it is recognized that the etiology of IR in PCOS is likely to be as elusive as in type 2 diabetes. Indeed, IR plays plays a major role in the metabolic and reproductive phenotypes of this syndrome. Insulin signaling in PCOS women may be as a result of the interaction of genetic and environmental factors that are specific to PCOS or T2DM [59]. Further studies on the effect of pharmacological and non-pharmacological approaches (e.g., physical exercise) in skeletal muscle of women with PCOS are of therapeutic relevance in this syndrome. 

## Figures and Tables

**Table 1:** Guidelines for the diagnosis of polycystic ovary syndrome.

NIH 1990^1^	Rotterdan 2003^2^	AES 2006^3^
Both criteria menstrual dysfunction	2 of the 3 criteria menstrual dysfunction	Both criteria menstrual dysfunction or polycystic ovary morphology
+	+	+
hyperandrogenemia or hyperandrogenism	hyperandrogenemia or hyperandrogenism	hyperandrogenemia or hyperandrogenism
	Polycystic ovary morphology	

+		
exclusion of other causes		
